# Tick-Borne Pathogens Screening Using a Multiplex Real-Time Polymerase Chain Reaction-Based Method

**DOI:** 10.1007/s11686-023-00702-0

**Published:** 2023-08-02

**Authors:** Sergio Andres Cardenas-Cadena, Maria Eugenia Castañeda-Lopez, Fabiana Esther Mollinedo-Montaño, Sodel Vazquez-Reyes, Jorge Lara-Arias, Ivan Alberto Marino-Martinez, Iram Pablo Rodriguez-Sanchez, Idalia Garza-Veloz, Margarita L. Martinez-Fierro

**Affiliations:** 1 Molecular Medicine Laboratory, Unidad Académica de Medicina Humana y Ciencias de la Salud, Universidad Autónoma de Zacatecas, Zacatecas, 98160 México; 2 Orthopedics and Traumatology Service, Facultad de Medicina y Hospital Universitario ‘Dr. José E. González’, Universidad Autónoma de Nuevo León, Monterrey, Nuevo León 64460 México; 3 Experimental Therapies Unit, Center for Research and Development in Health Sciences, Universidad Autónoma de Nuevo León, Monterrey, Nuevo León 64460 México; 4 Laboratory of Molecular and Structural Physiology, Facultad de Ciencias Biológicas, Universidad Autónoma de Nuevo León, Monterrey, Nuevo León 66455 México

**Keywords:** Tick-borne pathogens, Multiplex PCR, SYBR Green, PCR efficiency, Diagnostics

## Abstract

**Purpose:**

This study aims to develop and evaluate a cost-effective, user-friendly multiplex quantitative real-time polymerase chain reaction (qPCR) method for detecting multiple tick-borne pathogens associated with human and veterinary diseases.

**Methods:**

In silico PCR was performed to design and evaluate primer sequences reported for amplifying *Rickettsia spp*., *Borrelia spp.*, and *Ehrlichia spp.* Single and multiplex qPCR assays were then standardized to detect individual pathogens and multiple pathogens in a single reaction. Positive controls were generated to determine the dynamic range of the methods. In the validation phase, a total of 800 samples were screened for the presence of tick-borne pathogens.

**Results:**

Identification in a single qPCR reaction (multiplex) of *Ehrlichia spp.*, and *Borrelia spp.* with a limit of detection of 10 copies and *Rickettsia spp*. with 100 copies, a PCR efficiency (E) of 90–100% and a coefficient of correlation (*R*^2^) of 0.998–0.996 for all pathogens.

**Conclusion:**

The ability to detect three significant pathogens *(Ehrlichia spp*., *Rickettsia spp*., and *Borrelia spp*.) in a single qPCR reaction offers a significant advantage in the field of molecular diagnostics for tick-borne diseases. This advancement has a profound impact on public health as it facilitates the selection of appropriate treatment protocols, thereby reducing complications associated with disease progression. The streamlined approach provided by this method simplifies the diagnostic process and enables timely intervention, ultimately improving patient outcomes and mitigating the potential risks associated with untreated or misdiagnosed tick-borne infections.

**Supplementary Information:**

The online version contains supplementary material available at 10.1007/s11686-023-00702-0.

Ticks, belonging to two primary families, namely Argasidae (soft ticks) and Ixodidae (hard ticks), are ectoparasites that infest a wide range of terrestrial vertebrates, including certain amphibians and birds [1]. With approximately 867 tick species identified globally, spanning regions such as Asia [2], the Caribbean [3], Europe [4], Africa [5], South America [6], and Australia [7], nearly 10% of these species serve as vectors for an extensive array of pathogens. These pathogens are responsible for a diverse spectrum of diseases affecting both domestic animals and humans [8]. In the United States, tick-borne diseases exhibit a significant prevalence, with approximately 50,865 reported cases annually [9]. Among the bacterial diseases transmitted by ticks, the *Borreliaceae, Ehrlichiaceae,* and *Rickettsiaceae* families are of utmost concern [10]. *Borrelia burgdorferi* infection in humans is the leading cause of Lyme disease, however, in some cases, it has been associated with *Borrelia mayonii* and *Borrelia miyamotoi*, which are also found in black-legged tick (*Ixodes scapularis*) [11, 12]. Ehrlichiosis is a bacterial infection in humans caused by various species of *Ehrlichia* especially *E. chaffeensis, E. ewingii* and *Anaplasma (*formerly *Ehrlichia) phagocytophilum* which are primarily transmitted through the bite of infected ticks, such as the lone star tick (*Amblyomma americanum*) [13]. Different diseases related to the infection with rickettsia such as Rocky Mountain spotted fever (RMSF) are transmitted by the American dog tick (*Dermacentor variabilis*), Rocky Mountain wood tick (*Dermacentor andersoni*), and the brown dog tick (*Rhipicephalus sanguineous*) in the U.S [14]. Various diagnostic tools are available for diagnosing tick-borne diseases. These include serological tests such as ELISA, Western blot, and Indirect fluorescent antibody (IFA). It is important to note that these methods may exhibit limitations such as low sensitivity, potential cross-reactivity, or longer turnaround time, as seen in the case of bacterial culture. [15]. In contrast, molecular methods offer enhanced specificity, sensitivity, and rapidity for diagnostic test; in this sense, specific primers are generated to analyze conserved regions of DNA in genes like 16S rRNA and ompA of the target pathogen [16]. In this study, we have devised a novel approach using quantitative PCR (qPCR) to detect *Borrelia, Ehrlichia*, and *Rickettsia* pathogens by amplifying and detecting specific conserved regions of each organism. The development of a user-friendly diagnostic tool is imperative for strengthening disease prevention and control programs, as well as for providing precise and timely diagnosis crucial for effective treatment. For this purpose, we carefully selected genes and primer sequences that exhibit a close association with the respective bacterial families of concern. These primers were meticulously chosen and subjected to rigorous testing, focusing on conserved regions of the 16S rRNA gene for *Ehrlichia spp*. and *Borrelia spp*., and the ompA gene for *Rickettsia spp.* (See Table 1).

**Table 1:** General characteristics of the primers designed to amplify each target

Species	Target gene	Sequence of primers (5′-3′) *	Amplicon size (bp)	Reference
*Borrelia spp.*	16S rRNA	F-GCTGTAAACGATGCACACTTGGT R-GGCGGCACACTTAACACGTTAG	69	[28]
*Ehrlichia spp.*	16S rRNA	F-TCGCTATTAGATGAGCCTACGT R-GAGTCTGGACCGTATCTCAGT	124	[29]
*Rickettsia spp.*	OmpA	F-GGAGCGAATGCTGCACTAAT R-GTTCCGTTAATGGCAGCATCT	116	[23]

*All gene-specific primers synthetized and provided by T4 OLIGO® (Irapuato, Mex)

To confirm the cross-species amplification potential of selected primers, sequence homology analysis was performed using the Multiple Sequence Comparison by Log-Expectation (Muscle) tool from European Molecular Biology Laboratory (EMBL) and sequences from National Center for Biotechnology Information (NCBI) obtained via Basic Local Alignment Search Tool (BLAST) (Fig 1).

**Fig. 1:**
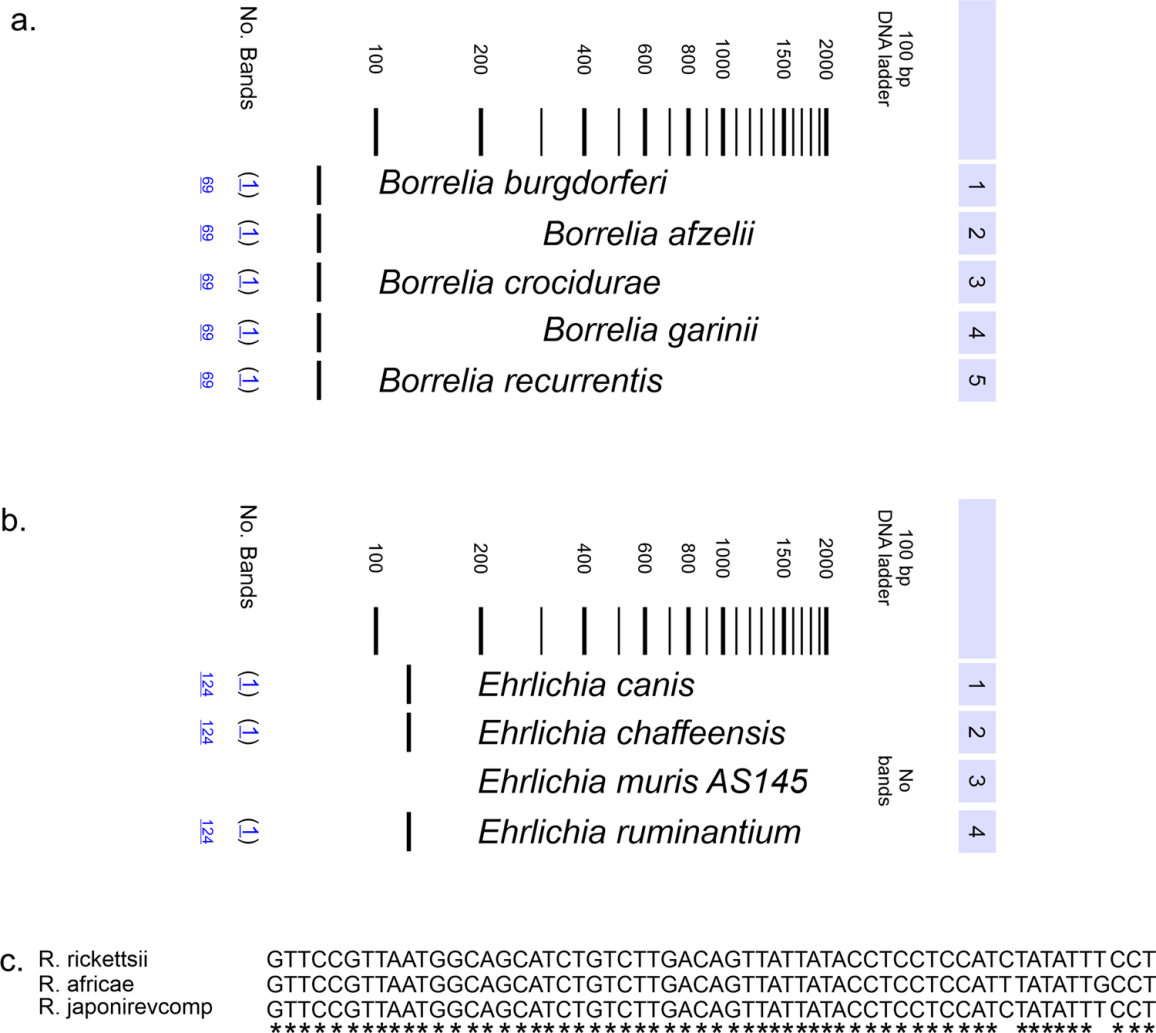
In silico analysis of primer sequences. Example of the in silico qPCR outcome format for a couple of primers targeting **A** *Borrelia spp*. **B** *Ehrlichia*. and **C** *Rickettsia spp*. genome sequences. Example of the in silico alignments of genome sequences in a conserved region of **D** *Borrelia spp*. **E** *Ehrlichia spp*. Using MUSCLE and **F** *Rickettsia spp*. Using BLAST

Positive qPCR controls for each target fragment were generated by cloning them into the pGEM-T Easy vector (Promega-Madison, U.S.A) and validated through sequencing. The detection limit [14] analysis involved constructing a standard curve comprising seven known concentration standards (STD) achieved through 1:10 serial dilution from the most concentrated STD (1,000,000 copies) to the most diluted STD (10 copies and one copy). The efficiency of the qPCR (E) and coefficient of correlation (*R*^2^) closely approached the desired values for a limit of detection of 10 copies STD, with *E = *90.33% and *R*^2^ = 0.998 for *Ehrlichia spp*, *E = *99.73% and *R*^2^ = 0.995 for *Borrelia spp,* and *E = *90% and *R*^2^ = 0.99 for *Rickettsia spp.* (Fig2). Co-infection controls were generated by all the combinations possible of positive controls and following the same parameters of STD preparation and evaluation the 1000 copies. STD results were *E = *112 to 75.3% and *R*^2^ > 0.9 (see supplementary Information S1).

**Fig. 2:**
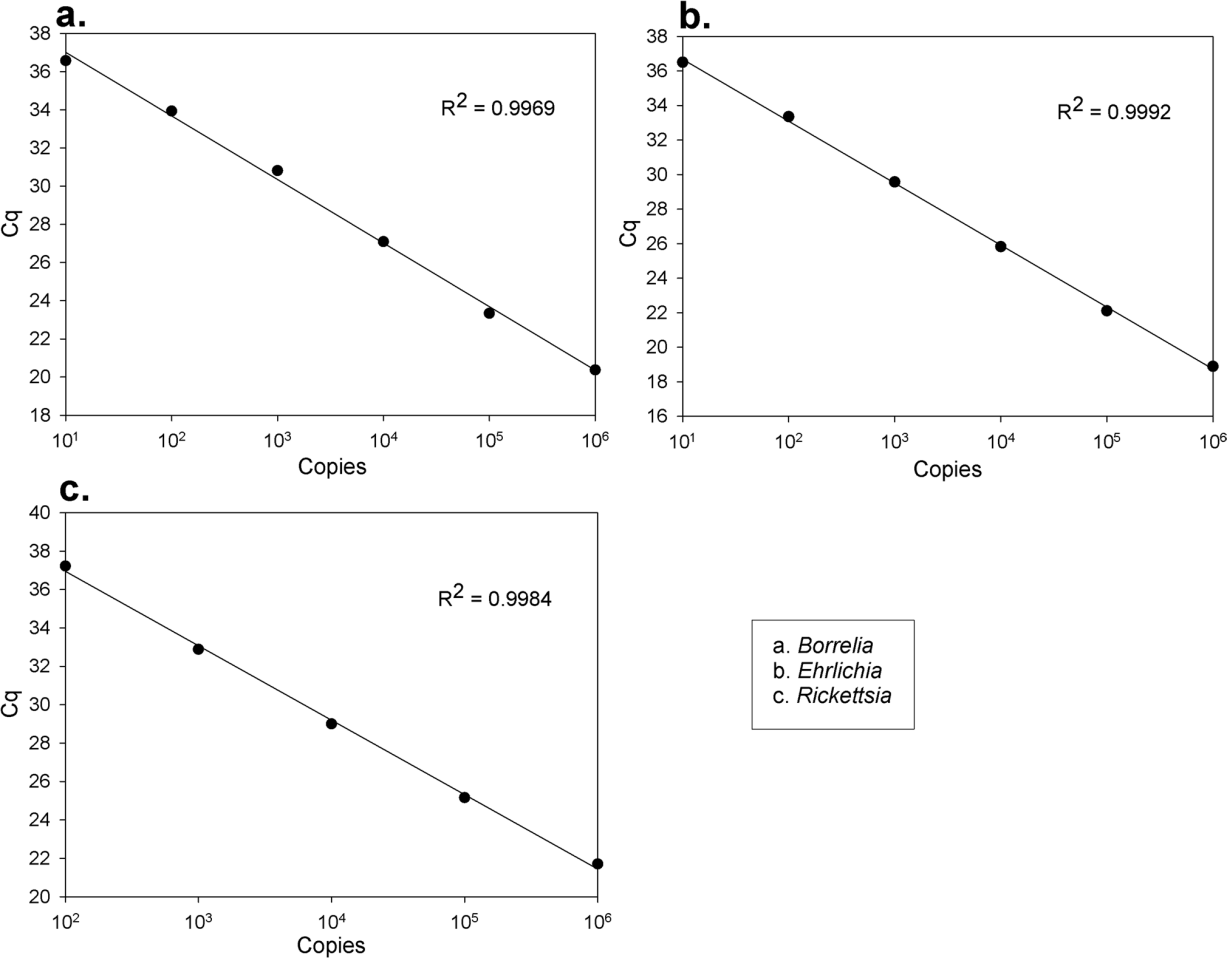
The detection limit of the target fragments by qPCR was analyzed for *Borrelia spp*. **A**, *Ehrlichia spp*. **B** and *Rickettsia spp*. **C**. The standard curves were analyzed using the SigmaPlot 11.0 software; the amplification efficiency was 90–100%, while the R^2^ was 0.99 on average for all the pathogens

To validate the multiplex qPCR method developed, 800 samples of DNA isolated from blood extracted from dogs (*n = *400) and ticks (*n = *400) were screened (Table 2). The sample status (positive or negative) was unknown prior to this test (see supplementary information S2).

**Table 2:** Summary of tick-borne pathogens screening in blood from dogs and tick samples

Pathogen	Positive DNA samples		Negative DNA samples		Samples screened	
	Dog (*n = *61)	Tick (*n = *254)	Dog (*n = *339)	Tick (*n = *146)	Dog (*n = *400)	Tick (*n = *400)
*Borrelia, n (%)*	14 (5)	21 (6)	286 (95)	329 (94)	300 (75)	350 (88)
*Ehrlichia, n (%)*	28 (7)	226 (57)	372 (93)	174 (43)	400 (100)	400 (100)
*Rickettsia, n (%)*	19 (6)	9 (3)	281 (94)	291 (97)	300 (75)	300 (75)

The Ehrlichiaceae family exhibited the highest prevalence among the analyzed samples, with 57% (226) in tick samples and 7% (28) in dog samples. Screening the samples using qPCR revealed that 5% of the tested samples were positive for at least one pathogen. Since these pathogens can co-infect the same host, it was necessary to create “co-infected” samples by mixing positive samples of different pathogens and standardize the qPCR to distinguish between them based on their characteristic signals. Notably, we successfully detected the “co-infection” of *Rickettsia spp* and *Borrelia spp*, making this assay the first multiplex SYBR-based test for detecting two out of three major tick-borne pathogens of human concern (refer to supplementary information S3). Details on the selection of study groups, bioethics, sampling, sample transportation, and DNA extraction can be found in supplementary information S2.

Collection and distribution of the blood samples obtained (dogs and ticks) are shown in Fig. 3.

**Fig. 3:**
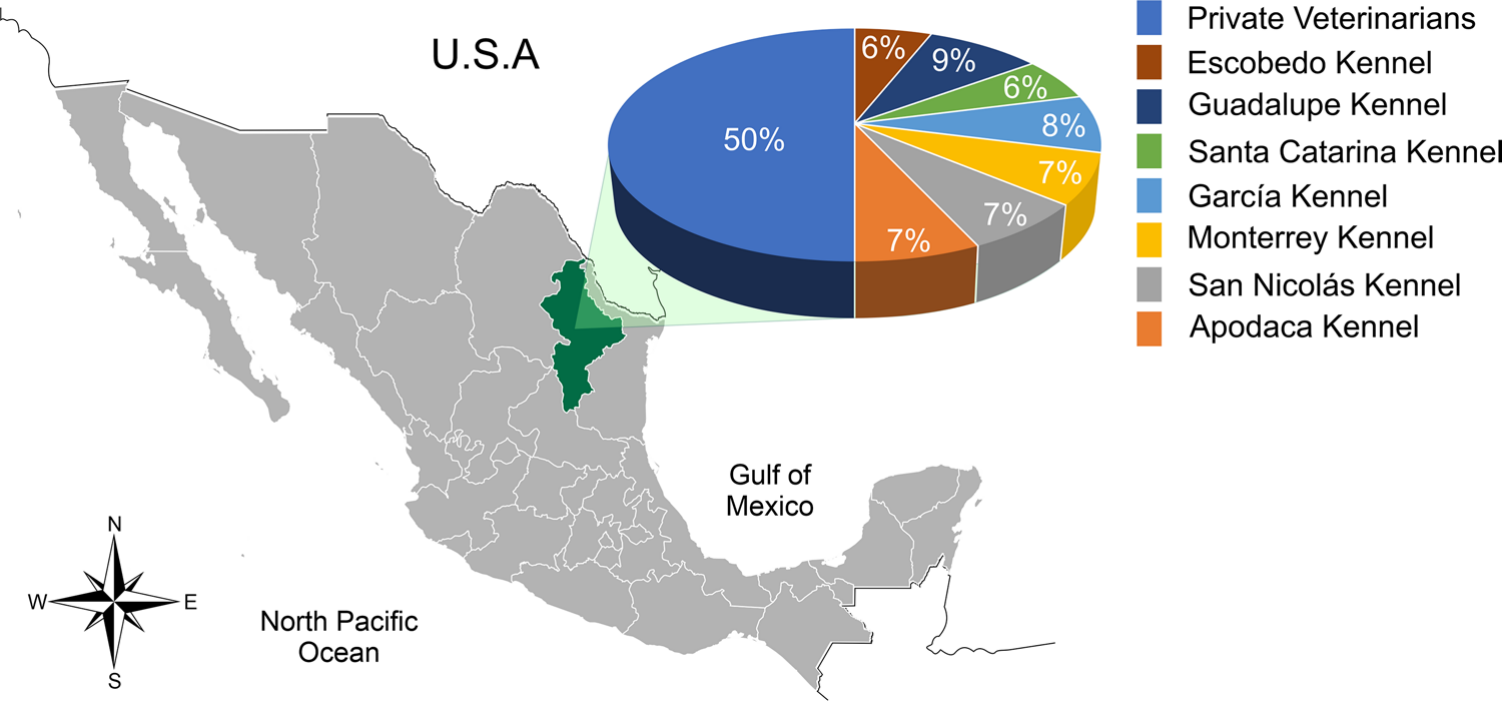
The 800 samples per collection center distribution shows that 50% of the samples were obtained in Mexican private clinics from Nuevo León State. The lowest proportion of samples was obtained in municipal kennels; the lowest percentage was Escobedo

Compared to other detection methods like TaqMan assays, our *Ehrlichia spp* detection sensitivity of ten copies was considerably good. TaqMan assays typically report detection limits of 5–50 copies [17–19]. Similarly, our *Borrelia spp* pathogen detection limit of ten copies aligns with the results of Saidac et al., who combined SYBR and TaqMan detection to achieve a quantification limit of 1 copy due to the low spirochete count [20]. Shan et al. achieved a detection limit of up to six copies in human DNA samples using TaqMan assays [21]. Our Rickettsia genus detection limits were tenfold below those of other studies, which detected at least five copies of the pathogen. Differences in assay type or amplicon size used in various studies may account for this variation [22, 23]. Our assay is less specific than the multiplex TaqMan assays which detect a lower number of copies, however, it remains having sensitivity and specificity above the gold standard ELISA or IFA technique, as the specificity of the proposed method remains above 99% for the pathogens evaluated [24, 25].

The intentional co-infection detected in this study involved mixing positive samples of Rickettsia and Borrelia genera in a single qPCR reaction. While no previous reports exist on this specific co-infection and its impact on the host, there have been reports of co-infections involving different Rickettsia spp., which can influence the transmissibility and disease dynamics caused by these pathogens [26]. Given the close phylogenetic relationship observed between *Ehrlichia chaffeensis* and *Anaplasma phagocytophilum*, our Ehrlichia primer set exhibits potential utility in a diagnostic PCR test for Ehrlichiosis caused by either of these two pathogens. In the validation results, Ehrlichia showed a higher prevalence compared to the other two pathogens in both tick and dog samples. This can be attributed to the strong affinity between *E. canis* and dogs. Notably, samples obtained from kennels yielded a higher number of positive samples, possibly due to the ease of transmission and the compromised health conditions typically found in such environments. While *Rickettsia* and *Borrelia* species were detected in smaller numbers, it is crucial to recognize that they remain significant health concerns, causing subclinical symptoms in infected dogs and in humans, these pathogens are responsible for several conditions including Rocky Mountain spotted fever and Lyme disease [4].

In this study, we have successfully developed the first effective and easy-to-use diagnostic tool using SYBR-based PCR technology, which despite being less specific, is still very sensitive and less expensive than TaqMan technology [27]. This tool enables the detection of three tick-borne pathogen families that are of significant clinical importance. By targeting conserved regions of the 16S rRNA and ompA genes, which are crucial in understanding the pathologies affecting both animals and humans, we have achieved the identification of multiple pathogens through a multiplex qPCR reaction. These findings serve as a foundation for future analyses, aiming to expand the scope of targets within the same diagnostic tool. The streamlined approach provided by this method simplifies the diagnostic process and facilitates timely intervention. By promptly identifying tick-borne infections, we can improve patient outcomes and mitigate the potential risks associated with untreated or misdiagnosed cases. The development of this diagnostic tool represents a significant advancement in the field, and we are optimistic about its potential to enhance diagnostic accuracy and improve patient care.

### Supplementary Information

Below is the link to the electronic supplementary material.

Supplementary file1 (DOCX 135 KB)

## Data Availability

All data supporting the reported results are included in the manuscript. Additional information regarding data that support the findings of this study will be available from the corresponding author upon reasonable request.
