# Correction: Tick-Borne Pathogens Screening Using a Multiplex Real-Time Polymerase Chain Reaction-Based Method

**DOI:** 10.1007/s11686-023-00785-9

**Published:** 2024-03-21

**Authors:** Sergio Andres Cardenas-Cadena, Maria Eugenia Castañeda-Lopez, Fabiana Esther Mollinedo-Montaño, Sodel Vazquez-Reyes, Jorge Lara-Arias, Ivan Alberto Marino-Martinez, Iram Pablo Rodriguez-Sanchez, Idalia Garza-Veloz, Margarita L. Martinez-Fierro

**Affiliations:** 1 Molecular Medicine Laboratory, Unidad Académica de Medicina Humana y Ciencias de la Salud, Universidad Autónoma de Zacatecas, 98160 Zacatecas, México; 2 Orthopedics and Traumatology Service, Facultad de Medicina y Hospital Universitario ‘Dr. José E. González’, Universidad Autónoma de Nuevo León, 64460 Monterrey, Nuevo León, México; 3 Experimental Therapies Unit, Center for Research and Development in Health Sciences, Universidad Autónoma de Nuevo León, 64460 Monterrey, Nuevo León, México; 4 Laboratory of Molecular and Structural Physiology, Facultad de Ciencias Biológicas, Universidad Autónoma de Nuevo León, 66455 Monterrey, Nuevo León, México

**Correction to: Acta Parasitologica (2023) 68:705–710**

10.1007/s11686-023-00702-0

The original version of this article, published on 02 August 2023, unfortunately contained a mistake. In this article ref. 28 was incorrect and should have been "Tsao JI, Wootton T, Bunikis J, Luna MG, Fish D, Barbour AG (2004), An ecological approach to preventing human infection: Vaccinating wild mouse reservoirs intervenes in the Lyme disease cycle. PNAS 101(52):18159–18164. 10.1073/pnas.0405763102"

The original article has been corrected.

