# Supplementary material for: Tick-Borne Pathogens Screening Using a Multiplex Real-Time Polymerase Chain Reaction-Based Method
Source: Acta Parasitol. 2023 Aug 2;68(3):705–10. doi: 10.1007/s11686-023-00702-0 (PMC10462521; doi:10.1007/s11686-023-00702-0)

**Tick-borne pathogens screening using a multiplex real-time polymerase chain reaction-based method**

**Acta parasitologica**

Sergio Andres Cardenas-Cadena^1†^, Maria Eugenia Castañeda-Lopez^1†^, Fabiana Esther Mollinedo-Montaño^1^, Sodel Vazquez-Reyes^1^, Jorge Lara-Arias^2^, Ivan Alberto Marino-Martinez^3^, Iram Pablo Rodriguez-Sanchez^4^, Idalia Garza-Veloz^1^, Margarita L Martinez-Fierro^1*^

^1^ Molecular Medicine Laboratory, Unidad Académica de Medicina Humana y Ciencias de la Salud, Universidad Autónoma de Zacatecas, Zacatecas 98160, México.

^2^ Orthopedics and Traumatology Service, Facultad de Medicina y Hospital Universitario ‘Dr. José E. González’, Universidad Autónoma de Nuevo León, Nuevo León 64460, México.

^3^ Experimental Therapies Unit, Center for Research and Development in Health Sciences, Universidad Autónoma de Nuevo León, Nuevo León 64460, México.

^4^ Laboratory of Molecular and Structural Physiology, Facultad de Ciencias Biológicas, Universidad Autónoma de Nuevo León, Nuevo León 66455, México.

† These authors contributed equally to this work.

*Corresponding author: [margaritamf@uaz.edu.mx](mailto:margaritamf@uaz.edu.mx); Tel.: +52 492 9256690 ext. 4535. ORCID: 0000-0003-1478-9068.

**SUPPLEMENTARY INFORMATION**

- Supplementary Information 1

S1: The amplification efficiencies (E) were 112.9%, 112.1%, 100.9% and 75.3% for *Ehrlichia spp./Borrelia spp.* (d), *Ehrlichia spp./Rickettsia spp.* (e), *Borrelia spp./Rickettsia spp.* (f) and *Ehrlichia spp./Borrelia spp./Rickettsia spp.* (g), respectively. While the R^2^ were close to 0.9 for all combination curves. All standard curves were analyzed in SigmaPlot 11.0 software.





- Supplementary Information 2

SI 2: Since in our study, we ensured that no additional procedures were conducted on the dogs, according to the Mexican guidelines NOM-029-ZOO-1995 “Characteristics and specifications for animal health testing and/or analysis laboratory facilities and equipment”, we did not need the involvement of a bioethics committee. We simply obtained the necessary permit from the dog owners to include their pets in our research. These dogs had visited the veterinarian due to symptoms suggestive of a potential tick-borne infection. If the veterinarian deemed a blood test necessary as part of their regular diagnostic procedure, we took the opportunity to explain our research project to the dog owners. We offered them the option to contribute to our study by redirecting a small portion (0.5mL) of the blood that would have otherwise been discarded into the regular veterinary diagnostic waste bin. All the sampling procedures was performed according to the Mexican guidelines NOM-029-ZOO-1995 “Characteristics and specifications for animal health testing and/or analysis laboratory facilities and equipment”.

In contrast, for study group 2 involving ticks, we obtained the necessary permit from the local authorities. The ticks were collected from street dogs by the dedicated staff at each anti-rabies center as part of their routine cleaning procedure. These ticks were generously donated to our research, with an average of 5 to 7 ticks per dog (400 dogs), which roughly equated to approximately 0.5 mL of blood for subsequent DNA extraction purposes.

Group 1 samples, consisting of dog blood, were collected in airtight blood tubes (Vacutainer, Becton, Dickinson and Company "BD," lavender top with EDTA). These tubes, containing 0.5 mL of blood per dog, were carefully transported in specialized coolers that maintained a controlled temperature range of 5°C to 15°C.

The extracted ticks, on the other hand, were placed in sterile 15 mL polypropylene conical centrifuge tubes (Thermo Scientific™), with each tube containing 5 to 7 ticks collected from a single dog. These tubes were transported at a temperature between 10°C and 20°C. Both sets of samples were processed in a biosafety level II laboratory (BSL II), adhering to all relevant biosafety regulations. DNA extraction and purification were carried out using the DNeasy Blood & Tissue Kits (Qiagen), following the manufacturer's instructions. Prior to DNA extraction, the samples from study group 2 (ticks) underwent a treatment involving mechanical maceration using a mortar and pestle (ensuring thorough cleaning between samples from different dog) and subsequent cell lysis with 180µL of ATL buffer and 20µL of Proteinase K per 50 mg of macerated tissue, mixed in a thermomixer at 56°C overnight. All reagents used for this process were from the same DNeasy Blood & Tissue Kits (Qiagen). Finally, all DNA samples were stored frozen at -20°C for future analysis.

- Supplementary information 3

S3: Detection of co-infection of *Borrelia spp*. and *Rickettsia spp*. by qPCR, which can be discriminated by melting curve. The melting curve temperature for *Rickettsia spp*. was 76.38° and for *Borrelia spp*. was 79.59°. The data were analyzed using StepOne v2.3 software.


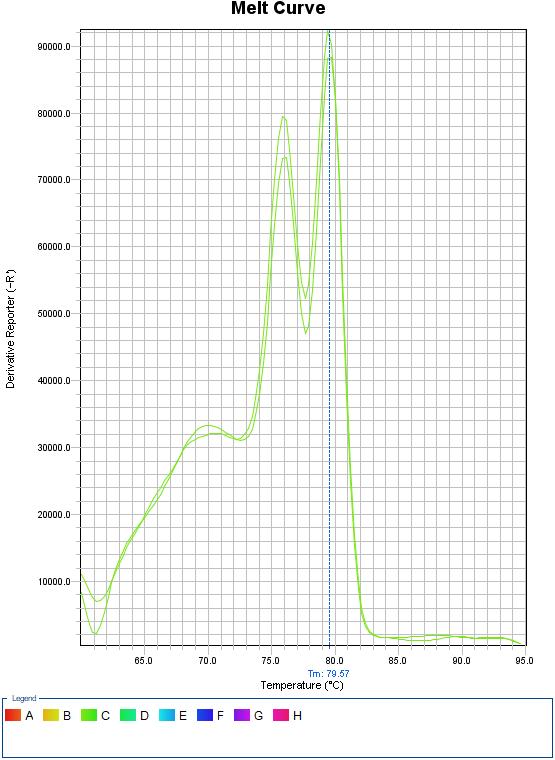

Supplement: Supplementary file 1 — Supplementary file1 (DOCX 135 KB) [file 11686_2023_702_MOESM1_ESM.docx]
